# Comparing real-life effectiveness of various COVID-19 vaccine regimens during the delta variant-dominant pandemic: a test-negative case-control study

**DOI:** 10.1080/22221751.2022.2037398

**Published:** 2022-02-16

**Authors:** Paskorn Sritipsukho, Thana Khawcharoenporn, Boonying Siribumrungwong, Pansachee Damronglerd, Nuntra Suwantarat, Araya Satdhabudha, Chanapai Chaiyakulsil, Phakatip Sinlapamongkolkul, Auchara Tangsathapornpong, Pornumpa Bunjoungmanee, Sira Nanthapisal, Chamnan Tanprasertkul, Naiyana Sritipsukho, Chatchai Mingmalairak, Anucha Apisarnthanarak, Pichaya Tantiyavarong

**Affiliations:** a Center of Excellence in Applied Epidemiology, Thammasat University, Pathumthani, Thailand; b Department of Pediatrics, Faculty of Medicine, Thammasat University, Pathumthani, Thailand; c Division of Infectious Diseases, Department of Internal Medicine, Faculty of Medicine, Thammasat University, Pathumthani, Thailand; d Department of Surgery, Faculty of Medicine, Thammasat University, Pathumthani, Thailand; e Department of Internal Medicine, Chulabhorn International College of Medicine, Thammasat University, Pathumthani, Thailand; f Department of Obstetrics & Gynecology, Faculty of Medicine, Thammasat University, Pathumthani, Thailand; g Thammasat University, Pathumthani, Thailand; h Department of Clinical Epidemiology, Faculty of Medicine, Thammasat University, Pathumthani, Thailand

**Keywords:** COVID-19, real-life, vaccine, effectiveness, delta variant, Thailand

## Abstract

Data on real-life vaccine effectiveness (VE), against the delta variant (B.1.617.2) of the severe acute respiratory syndrome coronavirus 2 among various coronavirus disease 2019 (COVID-19) vaccine regimens are urgently needed to impede the COVID-19 pandemic. We conducted a test-negative case-control study to assess the VE of various vaccine regimens for preventing COVID-19 during the period when the delta variant was the dominant causative virus (≥ 95%) in Thailand (25 July 2021–23 Oct 2021). All individuals (age ≥18 years) at-risk for COVID-19, presented for nasopharyngeal real-time polymerase chain reaction (RT-PCR) testing, were prospectively enrolled and followed up for disease development. Vaccine effectiveness was estimated with adjustment for individual demographic and clinical characteristics. Of 3353 included individuals, there were 1118 cases and 2235 controls. The adjusted VE among persons receiving two-dose CoronaVac plus one BNT162b2 booster was highest (98%; 95% confidence interval [CI] 87–100), followed by those receiving two-dose CoronaVac plus one ChAdOx1 nCoV-19 booster (86%; 95% CI 74–93), two-dose ChAdOx1 nCoV-19 (83%; 95% CI 70–90), one CoronaVac dose and one ChAdOx1 nCoV-19 dose (74%; 95% CI 43–88) and two-dose CoronaVac (60%; 95% CI 49–69). One dose of CoronaVac or ChAdOx1 nCoV-19 had a VE of less than 50%. Our study demonstrated the incremental VE with the increase in the number of vaccine doses received. The two-dose CoronaVac plus one BNT162b2 or ChAdOx1 nCoV-19 booster regimens was highly effective in preventing COVID-19 during the rise of delta variant.

## Introduction

The coronavirus disease 2019 (COVID-19) has become a global pandemic since early 2020. In Thailand, the third wave of the COVID-19 epidemic started in April 2021 and resulted in a significantly higher number of infected cases and mortality than those observed during previous epidemic waves. Most of the COVID-19 cases occurred in Bangkok and its vicinities. While the alpha variant (B.1.1.7) of severe acute respiratory syndrome coronavirus 2 (SARS-CoV-2) had rapidly spread during the early period, it has been almost completely replaced by the delta variant (B.1.617.2), which has now become the dominant virus causing COVID-19 in the country [1].

Mass vaccination programmes with highly effective vaccines are among strategies to impede the transmission of COVID-19. The vaccine effectiveness (VE) against wild-type or non-specified strains of SARS-CoV-2 infections were 47–66% for an inactivated viral vaccine [2,3], 62–71% for viral vector vaccines [4–6], and 91–93% for mRNA vaccines [7,8]. Concerns have been raised in regard to the lower VE against the delta variant than those against non-delta variants, including the alpha, beta or other non-specified variants (53–66% vs. 75–91%) [9–12], while the VE of BNT162b2 vaccine against delta variant infections was reported to be high during the first month (93%), but declined to 53% after 4 months [13]. To date, it is still unclear whether the reduced VE is due to waning immunity over time and/or the vaccine protection escape of the delta variant. In addition, there have been limited data on the VE of vaccine regimens other than the viral vector and mRNA vaccines, including those with a third-dose booster [14–16].

Vaccine demand that has surpassed supply, insufficient efficacy and adverse effect data, and differences in reported VE and the availability of COVID-19 vaccines have impacted mass vaccination programmes in each region. In Thailand, two-dose CoronaVac and two-dose ChAdOx1 nCoV-19 were the only two primary COVID-19 vaccines available in the early period (March 2021) of the programmes, while a limited number of BNT162b2 and BBIBP vaccines were subsequently available in August and September 2021. A rapid decline in immunity against SARS-CoV-2 during the first 3 months after completing the two-dose CoronaVac vaccine necessitates vaccination of a booster dose (as the third dose) of ChAdOx1 nCov-19 or BNT162b2 in the Thai population [17]. In the midst of the delta variant spread and the urgent need to achieve mass vaccination target (70% of population fully vaccinated), there was a shortage in ChAdOx1 nCov-19 supply in the country, and the requirement of a three-month interval between the two doses of ChAdOx1 nCov-19 made it difficult to reach the target in time to halt the epidemic. The heterologous CoronaVac/ChAdOx1 nCov-19 regimen vaccinated one month apart has been used since August 2021 based on its comparable immunogenicity to SARS-CoV-2 with the standard two-dose ChAdOx1 nCov-19 [18]. Although various COVID-19 vaccine regimens have been used in the country, there have been no studies evaluating the real-life VE against the delta variant.

## Methods

***Study design.*** We conducted a study in northern Bangkok and vicinities, Thailand, from 25 July 2021 to 23 October 2021. A test-negative case-control design was chosen to estimate the VE of COVID-19 vaccines given the ability to compare the VE of various regimens in the same platform and provide rapid results during the on-going epidemic [19]. In response to the COVID-19 epidemics, the Thai Government and Ministry of Public Health (MOPH) commenced the mass vaccination programmes in March 2021 and intensified the programme in June 2021. The vaccine regimens included two-dose CoronaVac (4 weeks apart between the two doses), two-dose BBIBP (4 weeks apart between the two doses), two-dose ChAdOx1 nCoV-19 (12 weeks apart between the two doses), one-dose CoronaVac and one-dose ChAdOx1 nCoV-19 (4 weeks apart between the two doses), two-dose CoronaVac plus one ChAdOx1 nCoV-19 booster (12 weeks apart between the second and booster doses) and two-dose CoronaVac plus one BNT162b2 booster (12 weeks apart between the second and booster doses). We compared vaccination status in individuals with confirmed COVID-19 with vaccination status in individuals who reported COVID-19 risks but had negative nasopharyngeal real-time polymerase chain reaction (RT-PCR) tests and no disease development after being followed up for 14 days. This study was approved by the Human Research Ethics Committee of Thammasat University (Medicine).

***Study population***. Adults ≥18 years old who are at risk for COVID-19 and presented to Thammasat University Hospital (TUH) and Thammasat Field Hospital (TFH) were eligible and approached for consent. The TUH is a 700-bed tertiary care medical centre serving the population in northern Bangkok and central Thailand, while TFH is a 490-bed field hospital branch of TUH, established for the comprehensive care of asymptomatic and mild COVID-19 patients. Inclusion criteria were individuals who met the country patients under investigation (PUI) criteria for COVID-19 (Supplementary Table 1) and received an initial nasopharyngeal RT-PCR test for SARS-CoV-2 at either facility. Exclusion criteria were those who did not consent to participate in the study or with known prior COVID-19 to estimate VE in fully susceptible persons.

### Study protocols and definitions

COVID-19 screening and management were according to the Thailand national guidelines on diagnosis, treatment and prevention of COVID-19 in healthcare facilities [20]. All PUI received initial nasopharyngeal RT-PCR tests within 5 days after the symptoms occurred or were suspected to have COVID-19 contacts. The 5-day duration was chosen based on the incubation period of the infections caused by the delta variant described in the previous study [21]. Subsequent nasopharyngeal RT-PCR tests were performed in those who later developed symptoms or had the initial test less than 5 days after COVID-19 contacts. Cases were defined as PUI whose initial or subsequent nasopharyngeal RT-PCR tests were positive, while controls were PUI with negative initial and all follow-up RT-PCR tests during the 14 days. All cases were categorized into asymptomatic, mild, moderate, severe, and critical diseases according to the World Health Organization criteria [22]. After the initial RT-PCR testing, controls were prospectively followed for symptom development using Chatbot software and telephone interview. Demographic data, comorbidities, COVID-19 case contact, and detailed history of COVID-19 vaccination, verified with the MOPH immunization centre, were collected using Research Electronic Data Capture (REDCap). We defined completion of each dose of vaccination as the receipt of the first, second and third doses for at least 21, 14 and 7 days at the time of enrolment, respectively. The definitions of vaccine completion for each dose were derived from the previous study of 2-dose vaccine regimens [23] and the study of the third booster dose regimen [14]. Data of causative variants of COVID-19 in the region were derived from the monthly reports of MOPH [1]. Comorbidities that increased risks for COVID-19 disease progression collected in this study included obesity (body mass index > 27 kg/m2), cardiovascular diseases, diabetes mellitus, chronic lung/airway diseases, chronic kidney diseases, malignancy, and cerebrovascular diseases. The primary outcomes were the VE in preventing COVID-19 regarding the number of doses and types of vaccine received, while the secondary outcomes were numbers of cases stratified according to disease severity and vaccine status.

***Statistical analysis.*** Categorical data were compared between cases and controls using the Chi-square test or Fisher’s exact test as appropriate. Based on the VE reported in the previous studies for CoronaVac and ChAdOx nCoV-19 vaccines [2,23], the estimated the VE of this study was 70%. To conduct a test-negative study with expected vaccination coverage of 30% for two major types of vaccine used in the country with a precision rate of ±10% and a 5% type I error rate, the required sample size was 900 for cases and 1800 for controls [24]. Logistic regression was used to estimate the odds of COVID-19 cases with a 95% confidence interval (CI) among vaccinated individuals compared with unvaccinated individuals. Vaccine effectiveness was (1-Odds ratio) ×100%. The multivariable regression model included covariates that could be associated with COVID-19. All significant factors with *P* < 0.05 from univariate analysis were included in the model to adjust confounding factors. The backward elimination method was used to select the final model. All analyses were performed using STATA, version 14 (StataCorp, College Station, Texas).

## Results

### Characteristics of the study population

**Figure 1.**
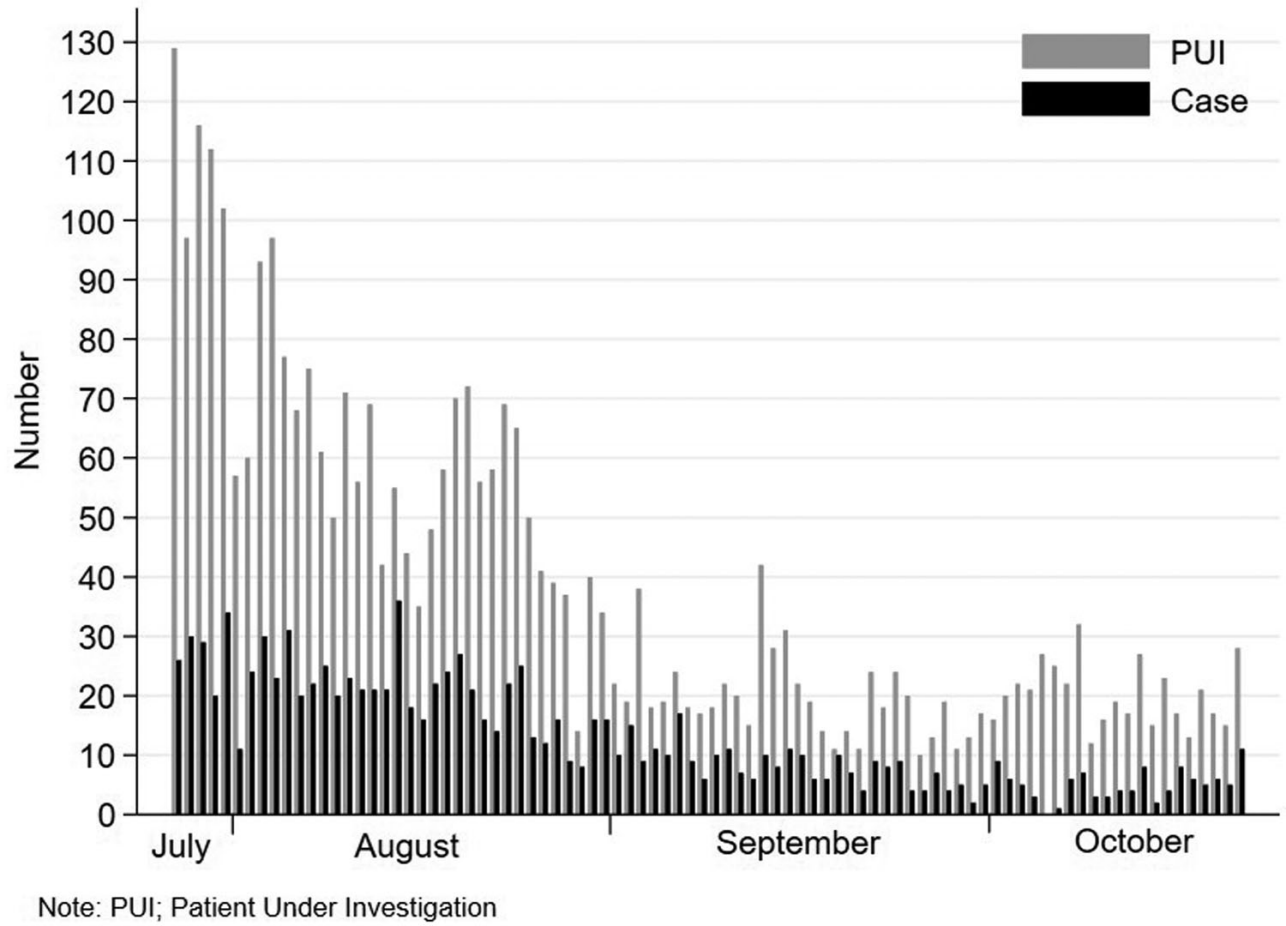
The distribution of patients under investigation (PUI) and cases during the study period.

Of the 3549 individuals meeting PUI criteria, 3353 (94.4%) consented to participate in the study, and none had prior COVID-19. The distribution of PUI and cases is shown in Figure 1. All these 3353 individuals (100%) completed the 14-day follow-up period. There were 1118 cases (33.3%) and 2235 controls (66.7%). Characteristics of the whole cohort, cases and controls are shown in Table 1. Cases were older and more likely to be male than controls. A significantly higher proportion of cases had lower education levels, had comorbidities and had contacted persons with COVID-19. In multivariable logistic regression analysis, independent factors associated with COVID-19 were age, sex, educational level, being a healthcare worker, and having any comorbidities at-risk for disease progression, while having persons with COVID-19 at home was not significantly associated with COVID-19.

**Table 1.** Characteristics of the study cohort, cases and controls.

Characteristic	All	Cases	Controls	*P* value^a^
	(*N* = 3353)	(*N* = 1118)	(*N* = 2235)	
Age (years)				<0.001
18–60	2958 (88.2)	913 (81.7)	2045 (91.5)	
>60	395 (11.8)	205 (18.3)	190 (8.5)	
Sex				<0.001
Male	1276 (38.1)	472 (42.2)	804 (40.0)	
Female	2077 (61.9)	646 (57.8)	1431(64.0)	
Highest education				<0.001
Primary school or less	542 (16.2)	276 (24.7)	266 (11.9)	
Secondary school	1184 (35.3)	502 (44.9)	682 (30.5)	
Bachelor degree	1396 (41.6)	296 (26.5)	1100 (49.2)	
More than Bachelor degree	231 (6.9)	44 (3.9)	187 (8.4)	
Occupation				<0.001
Healthcare worker	981 (29.3)	51 (4.6)	930 (41.6)	
Office worker	998 (30.0)	379 (33.9)	619 (27.7)	
Self-employed business	442 (13.2)	214 (19.1)	228 (10.2)	
School or college student	255 (7.6)	96 (8.6)	159 (7.1)	
Unemployed	677 (20.2)	378 (33.8)	299 (13.9)	
Comorbidities				
Obesity (BMI> 27 kg/m^2^)	833 (24.8)	323 (28.9)	510 (22.8)	<0.001
Cardiovascular diseases	466 (13.9)	205 (18.3)	261 (11.7)	<0.001
Diabetes mellitus	255 (7.6)	128 (11.5)	127 (5.7)	<0.001
Chronic lung/airway diseases	114 (3.4)	50 (4.5)	64 (2.9)	0.015
Chronic kidney diseases	45 (1.3)	13 (1.2)	32 (1.4)	0.523
Malignancy	45 (1.3)	21 (1.9)	24 (1.1)	0.056
Cerebrovascular diseases	27 (0.8)	9 (0.8)	18 (0.8)	1.00
Having persons with COVID–19 at home				<0.001
None	2143 (63.9)	616 (55.1)	1527 (68.3)	
1 person	806 (24.0)	291 (26.0)	515 (23.0)	
≥ 2 persons	404 (12.1)	211 (18.9)	193 (8.6)	
Prior rapid antigen test results^b^				<0.001
None	1843 (54.7)	429 (38.4)	1405 (62.9)	
Positive	553 (16.5)	496 (44.4)	57 (2.6)	
Negative	966 (28.8)	193 (17.3)	773 (34.6)	

Note: Data are in numbers (%).

^a^ Comparison between cases and controls.

^b^ Results of the latest nasal or nasopharyngeal rapid SARS-CoV-2 antigen tests within 72 h prior to nasopharyngeal real-time polymerase chain reaction tests.

Among 2311 individuals with initial negative RT-PCR test, repeated RT-PCR tests were performed in 462 (20.0%) [one time (*N* = 426, 18.4%) and two times (*N* = 36, 1.6%)]. The initial negative results converted to positive in 76 individuals. Of the 1118 cases, 67 (6.0%), 755 (67.5%), 226 (20.2%), 57 (5.1%) and 13 (1.2%) had asymptomatic, mild, moderate, severe and critical diseases, respectively. During the study period, the delta variant accounted for 95–100% of viruses causing COVID-19 in this region.

### Vaccine effectiveness estimates

**Figure 2.**
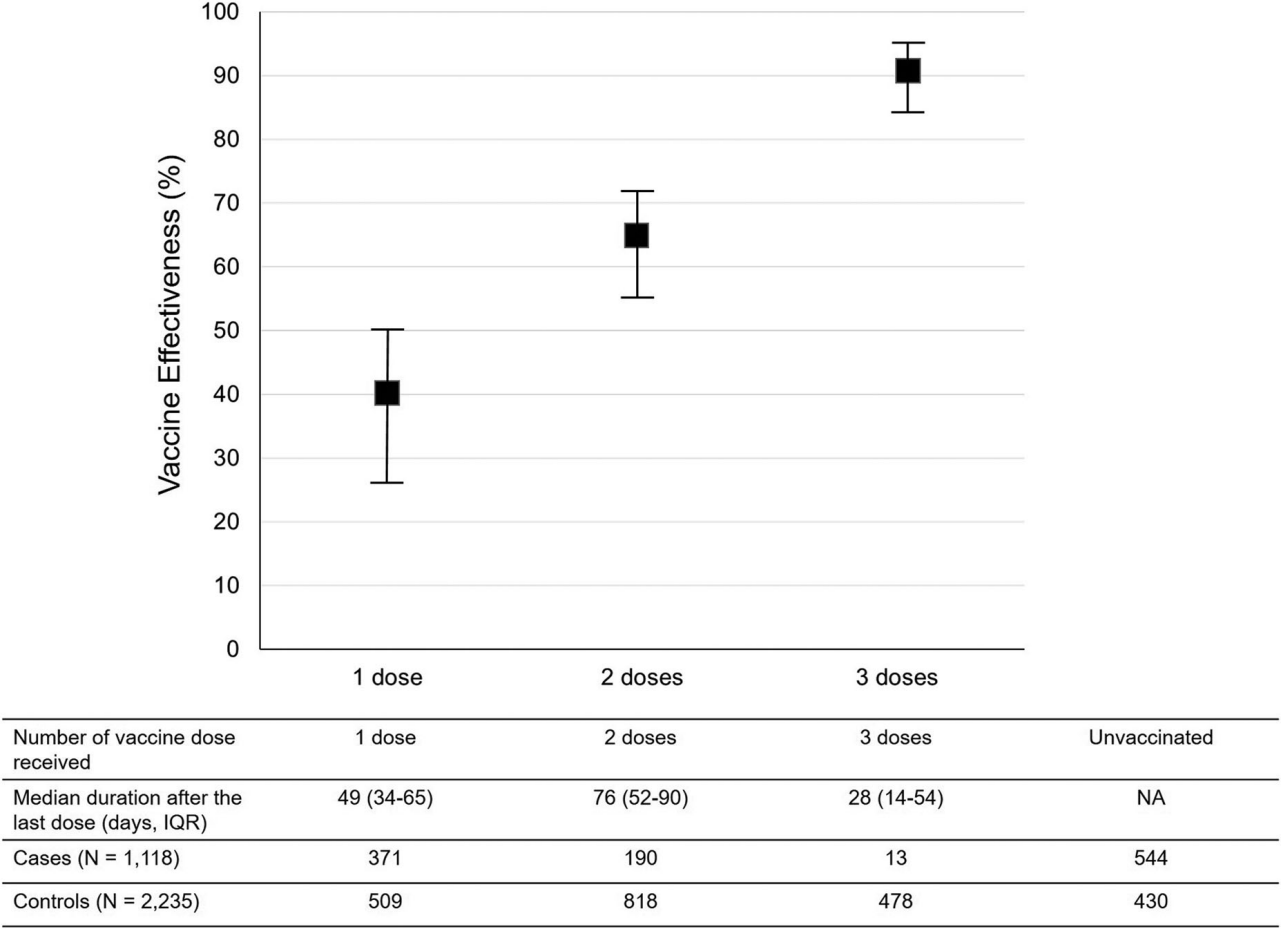
Vaccine Effectiveness according to the number of the dose received by the study participants. Notes: The VE was adjusted for being healthcare workers, comorbidities, age, educational level, and sex. I bars indicate 95% confidence intervals. IQR = interquartile range; NA = non-applicable.

Of the 2379 participants who received at least one dose of COVID-19 vaccines, 880 (37.0%), 1008 (42.4%) and 491 (20.6%) received one, two and three doses of the vaccines, respectively. In the analysis for “any vaccine” (Figure 2), the VE increased corresponding with the increase in the number of vaccine doses after adjustment for all independent factors associated with COVID-19. The VE among individuals who received one, two and three doses of the vaccines was 40% (95% CI, 27–50), 65% (95% CI, 56–72), and 91% (95% CI, 84–95), respectively.

**Figure 3.**
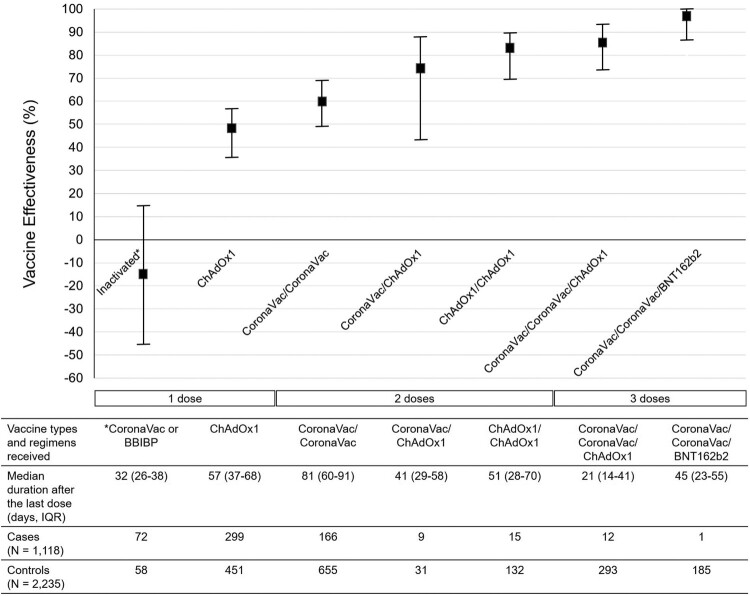
Vaccine effectiveness according to vaccine types and regimens received by the study participants. Notes: The VE was adjusted for being healthcare workers, comorbidities, age, educational level, and sex. I bars indicate 95% confidence intervals. IQR = interquartile range.

The three most common COVID-19 vaccine regimens received by the 2379 vaccinees at the enrolment were two-dose CoronaVac (*N* = 821, 34.5%), one-dose of ChAdOx1 nCoV-19 (*N* = 750, 31.5%), and two-dose CoronaVac plus one ChAdOx1 nCoV-19 booster (*N* = 305, 12.8%) (Figure 3). After multivariable adjustment for all independent factors associated with COVID-19, the VE among individuals receiving two-dose CoronaVac plus one BNT162b2 booster was highest (98%; 95% CI, 87–100), followed by those receiving two-dose CoronaVac plus one ChAdOx1 nCoV-19 booster (86%; 95% CI, 74–93), two-dose ChAdOx1 nCoV-19 (83%; 95% CI, 70–90), one-dose CoronaVac and one-dose ChAdOx1 nCoV-19 (74%; 95% CI, 43–88) and two-dose CoronaVac (60%; 95% CI, 49–69). The VE among those receiving only one dose of either CoronaVac or ChAdOx1 nCoV-19 was less than 50% (Figure 3). The distribution of participants receiving each vaccine regimen stratified according to disease status, and severity is shown in Table 2. Among the individuals receiving the three-dose regimens, all had asymptomatic or mild diseases.

**Table 2.** COVID-19 vaccination status of the study participants stratified according to diseases status and severity.

Vaccination status/Vaccine regimen	Total (*N* = 3353)	Controls (*N* = 2235)	Cases/Disease severity (*N* = 1118)				
			Asymptomatic	Mild	Moderate	Severe	Critical
			(*N* = 67)	(*N* = 755)	(*N* = 226)	(*N* = 57)	(*N* = 13)
Unvaccinated	974	430	36	349	115	37	7
1 dose							
CoronaVac or BBIBP	130	58	5	51	14	2	0
ChAdOx1	750	451	12	198	69	14	6
2 doses							
CoronaVac/CoronaVac	821	655	11	130	21	4	0
ChAdOx1/ChAdOx1	147	132	0	12	3	0	0
CoronaVac/ChAdOx1	40	31	0	5	4	0	0
3 doses							
CoronaVac/CoronaVac+ ChAdOx1 booster	305	293	3	9	0	0	0
CoronaVac/CoronaVac + BNT162b2 booster	186	185	0	1	0	0	0

Note: ChAdOx1 = ChAdOx1 nCoV-19 vaccine.

## Discussion

This study evaluated the real-life VE of COVID-19 vaccine regimens in the unique setting where inactivated and viral vector vaccines were the primary vaccines, and there was limited mRNA vaccine. In addition, the study was conducted during the time when there was an urgent need for the population to be fully vaccinated to combat the delta variant epidemic. The findings indicated that the VE increased significantly in correspondence with the increase in the number of received vaccine doses and one dose of either inactivated or vector viral vaccine had insufficient VE for preventing COVID-19. These findings were consistent with previous reports for inactivated vaccines in China [16] and mRNA vaccines in Norway and England [10,23]. However, a study from Qatar demonstrated a slight decrease in VE of mRNA vaccines after the second dose compared to after the first dose [13]. This was likely due to the waning immunity over time. Because, the VE was estimated after several months had passed since the second dose in a large proportion of the population in the study.

The VE against the delta variant after completing mRNA vaccines had dropped to 40–52% at 3 months after the second dose [25,26]. Given these findings and the surge of the delta variant pandemic, Israel, the US and the UK, have to recommend a third dose (or booster) of mRNA vaccines to people for whom 5 months have passed since their second dose. This recommendation has been supported by the recent findings, which revealed that giving a booster dose restored the VE on reducing breakthrough infection viral loads and risks for hospitalization, severe diseases and death [14,27]. In our study, we demonstrated the VE of 91% for the three-dose regimens (full-dose CoronaVac and a booster dose of ChAdOx1 nCoV-19 or BNT162b2), which was comparable to the VE of the three-dose BNT162b2 vaccine regimen reported previously (88%) [14]. To the best of our knowledge, this is the first study to report the real-life VE against delta variant of the heterologous third-dose boosting COVID-19 vaccine regimens and support their use in settings with limited viral vector and/or mRNA vaccine availability. The VE of the two-dose ChAdOx1 nCoV-19 vaccine was slightly lower than the three-dose regimens, and the two-dose CoronaVac vaccine had the lowest VE among full-dose vaccine regimens in this study. The lower VE of the two-dose inactivated vaccine was likely due to the less immunogenicity and rapid waning of the immunity within the first few months after full vaccination [17]. We found that the heterologous CoronaVac/ChAdOx1 nCoV-19 regimen had a considerably higher VE than the two-dose CoronacVac vaccine (74% vs. 60%). The higher VE of the heterologous than the homologous two-dose vaccine regimens may be explained by the higher immunogenicity and greater reactogenicity against SARS-CoV-2 induced by the heterologous regimens [28–30]. Altogether, our findings confirmed the improved VE against the delta variant using the heterologous two-dose inactivated/viral vector or three-dose inactivated/inactivated/viral vector or mRNA vaccine regimens.

The study’s strengths included the test-negative case-control design, which allows for the comparison of VE within the same healthcare facilities and minimizing the confounders from healthcare-seeking behaviours. The actual vaccine coverage rate of 20–40% during the study period was as anticipated for sample size calculation. The prospective collection of data and the 100% complete follow-up rate made it possible to obtain accurate baseline and follow-up variable data and minimize missing data and misclassification biases. In addition, the type, number of doses, and time of vaccine exposure used in this study were derived and validated from the national vaccine database. Nonetheless, there were some recognizable limitations. First, the study was conducted among participants who presented for COVID-19 screening in a single centre. The results may not be generalizable to other settings or population with no or limited healthcare access. Second, the causative variants of SARS-CoV-2 were not determined in each COVID-19 case. However, according to the MOPH SARS-CoV-2 variant surveillance report, we could assume that the delta variant had prevailed in the region during the study period. Third, the different VE may be subjected to the duration from the last vaccine dose received to the time of study enrolment. Lastly, since almost 30% of the study participants were HCWs who might have different risks of SARS-CoV-2 infections than the general population, some remaining confounding factors might affect the outcomes. However, the impact should be minimized since we used the multivariable logistic regression analysis to adjust all possible confounding variables when analysing the outcomes.

In conclusion, our study demonstrated the incremental VE with the increase in the number of received vaccine dose. Partial vaccination (one dose) had inadequate VE to prevent COVID-19 caused by the delta variant. The results confirm the adequate VE of the standard-dose ChAdOx1 nCoV-19 vaccine and suggest the need for heterologous vaccine boosting to improve VE after full-dose CoronaVac during the delta variant epidemic in Thailand. The heterologous CoronaVac/ChAdOx1 nCoV-19 regimen had high VE and may be used to shorten the time to complete vaccination among populations at-risk for COVID-19. Further studies are required to evaluate the real-life VE against the delta variant in other regions where the context of vaccine regimen and availability is different.

## Supplementary Material

Supplemental Material
